# Quantized conductance doubling and hard gap in a two-dimensional semiconductor–superconductor heterostructure

**DOI:** 10.1038/ncomms12841

**Published:** 2016-09-29

**Authors:** M. Kjaergaard, F. Nichele, H. J. Suominen, M. P. Nowak, M. Wimmer, A. R. Akhmerov, J. A. Folk, K. Flensberg, J. Shabani, C. J. Palmstrøm, C. M. Marcus

**Affiliations:** 1 Center for Quantum Devices and Station Q Copenhagen, Niels Bohr Institute, University of Copenhagen, Universitetsparken 5, 2100 Copenhagen, Denmark; 2 Kavli Institute of Nanoscience, Delft University of Technology, PO Box 4056, 2600 GA Delft, The Netherlands; 3 QuTech, Delft University of Technology, PO Box 4056, 2600 GA Delft, The Netherlands; 4 AGH University of Science and Technology, Faculty of Physics and Applied Computer Science, Mickiewicza 30, 30-059 Kraków, Poland; 5 Department of Physics and Astronomy, University of British Columbia, Vancouver, British Columbia, Canada V6T1Z1; 6 Quantum Matter Institute, University of British Columbia, Vancouver, British Columbia, Canada V6T1Z4; 7 California NanoSystems Institute, University of California, Santa Barbara, California 93106, USA; 8 Present address: Physics Department, City College of the City University of New York, New York 10031, USA

## Abstract

Coupling a two-dimensional (2D) semiconductor heterostructure to a superconductor opens new research and technology opportunities, including fundamental problems in mesoscopic superconductivity, scalable superconducting electronics, and new topological states of matter. One route towards topological matter is by coupling a 2D electron gas with strong spin–orbit interaction to an s-wave superconductor. Previous efforts along these lines have been adversely affected by interface disorder and unstable gating. Here we show measurements on a gateable InGaAs/InAs 2DEG with patterned epitaxial Al, yielding devices with atomically pristine interfaces between semiconductor and superconductor. Using surface gates to form a quantum point contact (QPC), we find a hard superconducting gap in the tunnelling regime. When the QPC is in the open regime, we observe a first conductance plateau at 4*e*^2^/*h*, consistent with theory. The hard-gap semiconductor–superconductor system demonstrated here is amenable to top-down processing and provides a new avenue towards low-dissipation electronics and topological quantum systems.

## Abstract (web summary)

Interface transparency between 2D semiconductors and superconductors is a longstanding problem, seriously hindering potential applications. Here, using a new hybrid system, Kjaergaard *et al*. report quantized conductance doubling and a hard superconducting gap measured via a quantum point contact, indicating a near pristine interface.

## Graphical abstract

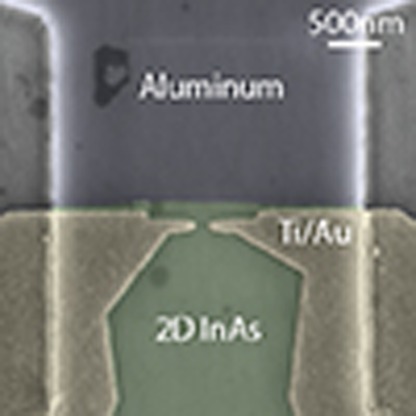

Recent work on semiconductor nanowires has offered evidence for the existence of Majorana zero modes, a signature of topological superconductivity^1^,^2^,^3^. A characteristic of the first studies in this area was significant subgap tunnelling conductance (a so-called soft gap), attributed to disorder at the semiconductor–superconductor (Sm–S) interface^4^,^5^. In nanowires, the soft-gap problem was recently resolved by growing Al epitaxially on InAs nanowires, yielding greatly reduced subgap conductance^6^,^7^. Studies of Sm–S systems based on top-down processed gateable two-dimensional electron gases (2DEGs) coupled to superconductors have not explicitly addressed the soft-gap issue yet^8^,^9^. However experiments on such systems have demonstrated other theoretical predictions, such as quantization of critical current^9^,^10^,^11^, the retro-reflection property of Andreev scattering^12^, and spectroscopy of a gate-defined quantum dot with superconducting leads^13^,^14^, which do not require a hard proximity-induced gap in the semiconductor.

The two main results we present in this paper are both consequences of the transparent epitaxial Sm–S interface and overcome the soft gap problem for 2D electron gases. The first is a doubling of the the lowest quantized conductance plateau, from 2*e*^2^/*h* in the normal state to 4*e*^2^/*h* in the superconducting state, as predicted theoretically^15^. The second is a strong suppression of conductance for voltages smaller than the superconducting gap when the quantum point contact (QPC) is in the tunnelling regime—that is, the detection of a hard superconducting gap in a proximitized 2DEG. Conductance doubling arises from Andreev reflection transferring charge 2*e* into the superconductor^16^. The hard gap reflects the absence of electronic states below the superconducting gap in the semiconductor. Using gate voltage to control the QPC, we measure conductance across the transition from weak tunnelling to the open-channel regime and find good (but not perfect) agreement with the theory of a normal-QPC-superconductor structure^15^.

## Results

### Properties of the 2DEG and the superconducting Al film

**Figure 1:**
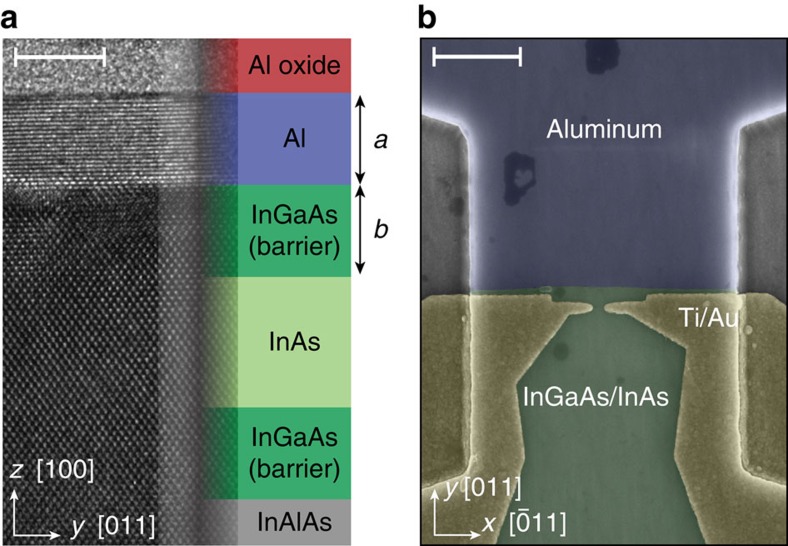
Epitaxial aluminium on InGaAs/InAs and device layout. (**a**) Cross-sectional transmission electron micrograph of epitaxial Al on InGaAs/InAs. On the wafer imaged here, the height of the InGaAs barrier is *b*=5 nm and Al film thickness *a*∼5 nm. Scale bar, 5 nm. (**b**) False-colour scanning electron micrograph of Device 1 (see main text for details). Scale bar, 1 μm.

The starting material is an undoped InAs/InGaAs heterostructure with epitaxial Al as a top layer, grown by molecular beam epitaxy^17^. A cross-sectional TEM showing a sharp epitaxial Sm–S interface is shown in Fig. 1a. In the devices reported here, the thickness of the InGaAs barrier was *b*=10 nm, and the Al film thickness was *a*=10 nm. A Hall ball fabricated on the same wafer with the Al removed (see Methods) gave density *n*=3 × 10^12^ cm^−2^ and mobility *μ*=10^4^ cm^2^ V^−1^ s^−1^, yielding a mean free path *l*_e_∼230 nm. In a similar wafer, weak anti-localization analysis gave a spin–orbit length *l*_so_∼45 nm (ref. 17). The Al film has a critical temperature *T*_c_=1.56 K, corresponding to a gap Δ_0_=235 μeV, enhanced from the bulk value of Al, and consistent with other measurements on Al films of similar thickness^18^. The in-plane critical field of the Al film is *B*_c_=1.65 T (ref. 17).

### Quantized conductance doubling

**Figure 2:**
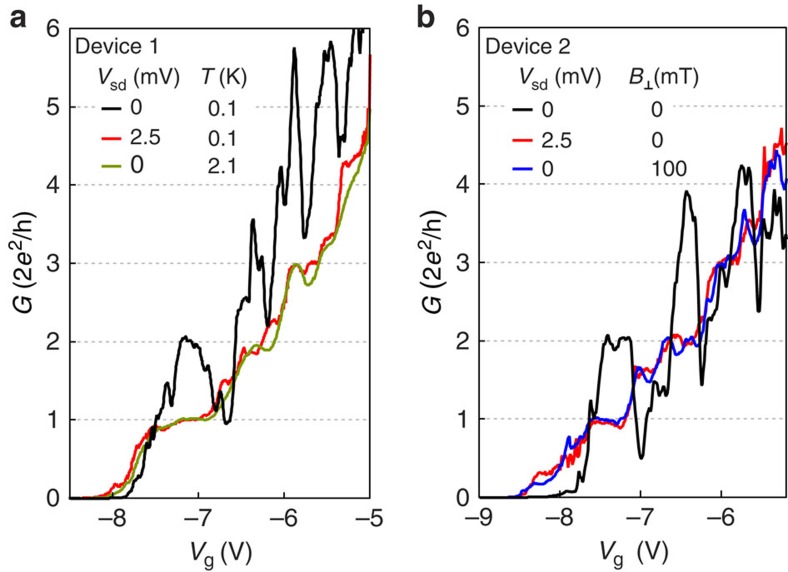
Quantized conductance in the Andreev quantum point contact. (**a**) Differential conductance, *G*, as a function of gate voltage *V*_g_ at zero bias (black line), at source-drain bias larger than the gap (red line), and at elevated temperature (green line). At zero bias and base temperature, the first conductance plateau is at 4*e*^2^/*h*, double the value at higher temperature or bias. (**b**) The differential conductance in a second, lithographically identical device at zero bias (black line), at source-drain bias larger than the gap (red line), and in a magnetic field applied perpendicular to the plane of the chip (blue line).

A scanning electron micrograph of Device 1 is shown in Fig. 1b. The conductance of the QPC is tuned by negative voltages applied to the gates. The QPC is located ∼150 nm in front of the region where the Al film has not been removed. Figure 2 shows conductance traces for two lithographically similar QPCs. In the superconducting state, both devices show increased conductance at the plateau of the QPC and suppressed conductance below *G*∼0.8*G*_0_, where *G*_0_≡2*e*^2^/*h*, relative to the normal state. This behaviour is the hallmark of Andreev reflection being the dominant conduction mechanism through the QPC^15^,^19^. Raising the temperature above the critical temperature of the Al film, applying an out-of-plane magnetic field, or applying a bias larger than the gap, all bring the lowest plateau back to 2*e*^2^/*h* (Fig. 2). The dip structure at the transition between conductance plateaus was also observed in a similar experiment on nanowires^20^, and is presumably caused by mode mixing due to disorder, leading to a reduction in transparency of the already open first channel. A constant contact resistance *R*_c_∼1 kΩ has been subtracted in each viewgraph, a value chosen to move the first plateau in the normal state to *G*_0_.

### Hard superconducting gap

**Figure 3:**
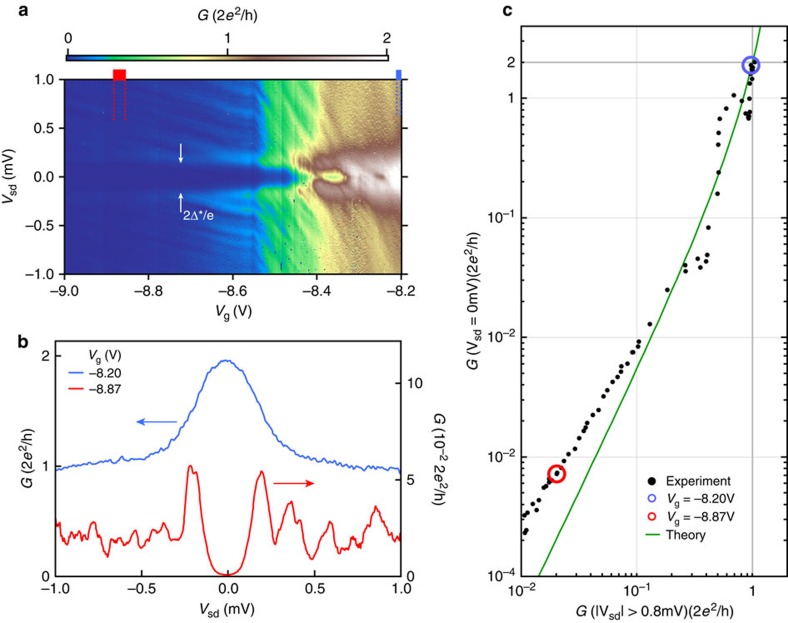
Transition from 4*e*^2^/*h* conductance to hard superconducting gap. (**a**) Differential conductance, *G*, in Device 1 as a function of gate voltage *V*_g_ and source-drain voltage bias *V*_sd_. (**b**) Vertical cuts in **a** in the tunnelling (red line) and one-channel (blue line) regime. Supplementary Figure 1 shows data from a lithographically similar device on a wafer with no InGaAs barrier (that is, *b*=0 nm) between the top layer Al and the InAs 2DEG. (**c**) Differential conductance at zero source-drain voltage, *G*(*V*_sd_=0 mV), versus averaged differential conductance at finite source-drain voltage, *G*(|*V*_sd_|>0.8 mV). Red and blue circles indicate data corresponding to cuts in **b**. Green line is the theoretically predicted conductance in an Andreev enhanced QPC (equation (1) with no fitting parameters).

By further depleting the electron gas in the constriction, the device is operated as a tunnel probe of the local density of states in the InAs 2DEG. This technique has been applied to studying subgap properties of semiconductor nanowires coupled to superconductors^1^,^2^,^3^,^6^,^21^,^22^. In Fig. 3a, the QPC voltage is decreased to gradually transition from the one-channel regime, where the zero bias conductance is 4*e*^2^/*h*, to the tunnelling regime, where conductance is strongly suppressed for |*V*_sd_|<190 μV. From these measurements, the gap in the density of states of the InAs due to the proximity to the Al is estimated to be Δ*∼190 μeV (measured peak-to-peak). The value of Δ* is similar, but not identical, to the gap in the Al film as estimated from *T*_c_, as discussed above.

In the case of perfect Andreev reflection from the superconductor/semiconductor interface, the conductance of one channel through a constriction proximal to the interface is given by


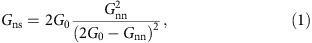


where *G*_ns_ is the conductance when the film is superconducting, and *G*_nn_ is the conductance in the normal state^15^. In Fig. 3c, the prediction in equation (1) with no free parameters (green line) and experimental data are shown. Here, *G*_nn_ is the average conductance for |*V*_sd_|>0.8 mV, justified by the equality of applying a bias and raising the temperature above *T*_c_, as shown in Fig. 2a. Equation (1) is consistent with the data over two orders of magnitude in *G*_ns_, indicating that the zero bias conductance up to 4*e*^2^/*h* is well described by the prediction of perfect Andreev reflection of a single QPC mode. Equation (1) represents the only quantitative theory of the relation between subgap conductance and normal state conductance (that is, the hard gap) of which we are aware, and the agreement between equation (1) and the experiment in Fig. 3c leads to the designation of a hard gap in this superconductor–2DEG system. However, the systematic deviation between data and prediction in Fig. 3c for *G*_ns_<10^−2^ × 2*e*^2^/*h* could be a manifestation of a small remnant non-zero normal scattering probability.

The shapes of the conductance curves at *eV*_sd_≲Δ* in the tunnelling regime (red line in Fig. 3b) are smeared relative to the conventional Bardeen–Cooper–Schrieffer (BCS) density of states of a superconductor. This could be due to broadening of the BCS coherence peaks in the disordered superconducting film formed in the 2DEG under the Al^23^, a weak coupling between Al and 2DEG^5^ or the layout of the tunnel probe relative to the proximitized 2DEG^24^,^25^,^26^.

### Temperature dependence of the density of states

**Figure 4:**
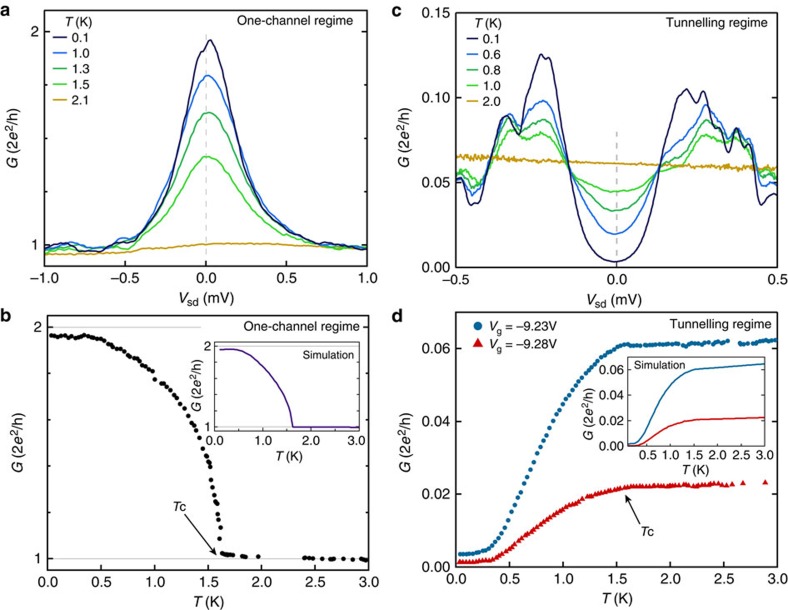
Temperature dependence of the enhanced subgap conductance and the hard superconducting gap. (**a**) Differential conductance, *G*, as a function of source-drain bias voltage, *V*_sd_, at five temperatures in the one-channel regime. See Supplementary Fig. 2a for similar data measured on a wafer with no InGaAs barrier between the top layer Al and the InAs 2DEG. (**b**) Temperature dependence at zero bias (corresponding to cut along the dashed, grey line in **a** in the one-channel regime. (**c**) Similar measurement to **a** but in the tunnelling regime. (**d**) As in **b** for two different values of gate voltage, *V*_g_, both in the tunnelling regime. Insets in **b** and **d** show results from numerical simulations (see Supplementary Figs 3–5 for more details on numerical results).

The temperature dependence of the conductance in the Andreev QPC is different in the one-channel and in the tunnel regime (Fig. 4). The one-channel regime (Fig. 4a,b) has a pronounced kink at *T*=*T*_c_, presumably associated with the sudden onset of Andreev enhanced subgap conductance. In contrast, the temperature dependence in the tunnel regime (Fig. 4c,d) is smeared close to *T*_c_ due to thermally excited quasiparticles.

The temperature dependence is simulated (insets in Fig. 4) by calculating 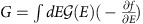 where *f* is the Fermi function that accounts for thermal broadening. The conductance 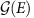 is calculated by combining scattering matrices of electrons and holes in the normal region and Andreev reflection at the superconductor interface (details of the simulation are given in Methods). The scattering matrices are calculated using the numerical package Kwant^27^, and the simulation are performed using the device geometry from the micrograph in Fig. 1b. The temperature dependence of the gap is modeled with 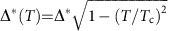, and the Andreev reflection amplitude is taken from ref. 15. The simulation shows good quantitative agreement with the data.

### Magnetic field dependence of the density of states

**Figure 5:**
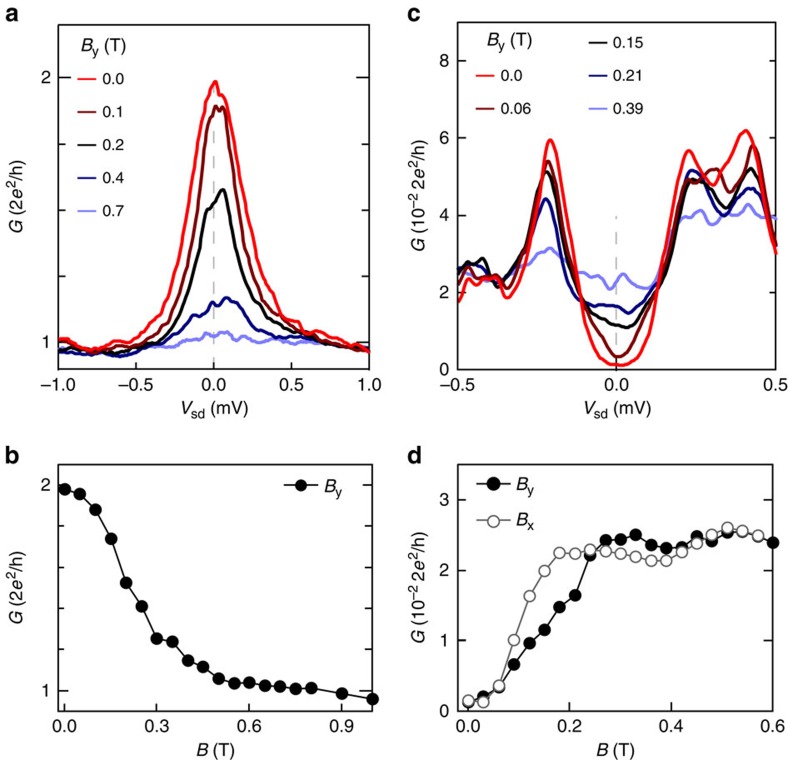
In-plane magnetic field of the enhanced subgap conductance and the hard superconducting gap. (**a**) Differential conductance, *G*, as a function of source-drain bias, *V*_sd_, at several in-plane magnetic fields applied along the point contact constriction. (**b**) Zero-bias conductance as a function of the in-plane magnetic field, *B*_*y*_. (**c**) Similar measurement to **a** but in the tunnelling regime. Supplementary Fig. 2b shows data on a lithographically similar device fabricated on a wafer with no InGaAs barrier between the top layer Al and InAs 2DEG (that is, *b*=0 nm). (**d**) As in **b** but in the tunnelling regime, for both in-plane directions (*B*_*y*_ is along and *B*_*x*_ is perpendicular to the constriction).

To drive a superconductor/semiconductor device into a topological regime, one requirement is *gμ*_B_*B*>Δ*, while the native superconductor retains its gap. Figure 5 shows the in-plane magnetic field dependence of Δ*, from which an approximate critical field 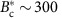 mT is extracted. A rough estimate of the *g*-factor can be inferred by assuming the critical 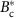 results from Zeeman energy surpassing the induced superconducting gap, that is 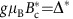, which yields *g*∼10, similar to the *g*-factor in bulk InAs. In Fig. 5d, the zero-bias conductance is shown for the two different in-plane directions, and the slight direction dependence of 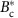 could be due to an anisotropic *g*-factor in the InAs crystal lattice. The induced gap in the 2DEG disappears at in-plane magnetic fields significantly smaller than the critical field of the Al film itself. The 2DEG has a strong spin–orbit interaction (*l*_so_∼45 nm), which, taken together with the intimate coupling to the superconductor, makes this material system a feasible candidate to realize topological superconducting devices. By using top-down fabrication techniques and the electrostatic gating demonstrated here, effective one-dimensional systems can be produced, in which an in-plane magnetic field can close the induced superconducting gap to reach a topological phase.

In conclusion, we observe quantization doubling through a QPC proximal to a superconductor/semiconductor interface, confirming a long-standing theoretical prediction^15^. Operated as a gate-tunable tunnel probe of the local density of states, the QPC shows a hard superconducting gap induced in the 2DEG. The magnetic field dependence of the induced gap compares favourably with the critical field of the superconducting film, opening possibilities to pursue topological states of matter in one-dimensional structures fabricated from epitaxial Al/2D InAs material.

## Methods

### Fabrication and measurement setup

Ohmic contacts to the InAs electron gas are formed directly by the epitaxial Al. Mesa structures are patterned by standard III–V chemical etching techniques. The aluminium is etched using commercial Transene Aluminum Etch D. Subsequent to the selective Al etch, an insulating 40 nm Al_2_O_3_ layer is deposited using atomic layer deposition and metallic gates (5 nm Ti/50 nm Au) are evaporated onto the device. The measurements were performed in a dilution refrigerator with a base-mixing chamber temperature *T*_mc_∼30 mK, using four-terminal lock-in techniques and DC measurements.

### Measurement details

The data in Fig. 3 is measured in a DC setup, incrementing the voltage in steps of size 3 μV. The data are smoothed over 10 steps and the derivative is calculated numerically to obtain the differential conductance. A constant contact resistance *R*_c_=800 Ω is subtracted from the data, moving the conductance at *V*_g_=−8.2 V for *V*_sd_>0.8 mV to 2*e*^2^/*h*. The four-terminal resistance of the device is *R*_d_=400 Ω with *V*_g_=0 V. The difference between *R*_c_ and *R*_d_ is most likely dominated by the change of resistivity near the gated region, when the gate is turned on, as well as the distance from the voltage probe to the QPC region. The voltage probes are located ∼15 μm away from the QPC and the gates overlap the mesa over an area ∼1.6 μm^2^. The normal state conductance is calculated as the average of *G*(*V*_sd_) for *V*_sd_ in the range (±0.8 mV, ±1 mV). The analysis is largely unaffected by changing the averaging window for values |*V*_sd_|>0.6 mV. The cuts in Fig. 3b are taken by averaging over a 12 mV (30 mV) window in *V*_g_ for the one-channel (tunnelling) regime. Finally, each datapoint in Fig. 3c is calculated as the average over a 10 mV range in *V*_g_.

### Model for numerical simulations

We calculate the conductance of the junction in two steps. First, we determine the scattering properties of the normal region which we assume is a 1.1 μm wide channel of length *L*, where we have taken dimensions from SEM in Fig. 1b. It is described by the spinless Hamiltonian,


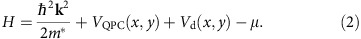


We model the QPC as two rectangular gates located at *X*=400 nm, with the width 2*W*, separated by the length 2*S* and located at the distance *d* above the 2DEG (see Supplementary Fig. 3 for illustration of *W* and *S*). We calculate the potential generated by the QPC electrodes, *V*_QPC_(*x*, *y*), for the gate voltage *V*_g_ as follows^28^


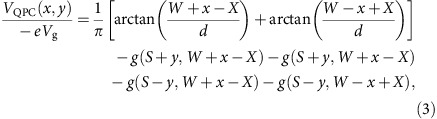


where


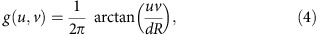


and 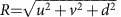. The potential landscape of the simulation is shown in Supplementary Fig. 3.

We include disorder^29^ by adding a random on-site energy *V*_d_(*x*, *y*) distributed uniformly between −*U*_d_/2 and *U*_d_/2 where


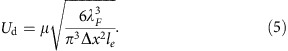


Due to limitation of the computational mesh resolution we exclude the disorder from the vicinity of the QPC and take *U*_d_≠0 only for *x*>700 nm.

We calculate the scattering matrix of the normal part of the junction for a particle at the energy *ɛ* as


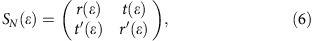


using Kwant package^27^ and discretizing the Hamiltonian in equation (2) on a mesh with the spacing Δ*x*=Δ*y*=3 nm. The quantities *r*(*ɛ*) and *t*(*ɛ*) denote reflection and transmission submatrices for a time-reversal symmetric system. In the second step, we combine the scattering matrices calculated for *ɛ* and −*ɛ* (that correspond to electron and hole, respectively) with the matrix that accounts for the Andreev reflection at the superconductor interface


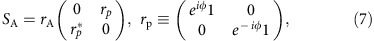


where


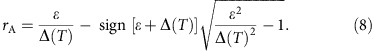


The latter equation describes the Andreev reflection amplitude^15^ including the temperature-dependent pairing potential 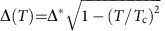. Finally, we calculate the conductance according to


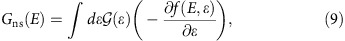


where *f* stands for the Fermi function


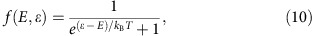


and where 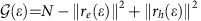. *N* is the number of modes in the normal lead. The quasielectron and quasihole reflection matrices are given by:


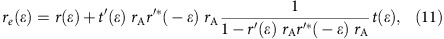



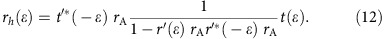


Additionally, the normal-state conductance is given by 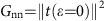. Results of the simulations are shown in Supplementary Figs 3–5.

### Data availability

All data presented in the main paper and supplement, as well as code used to generate simulations are available from the authors upon request.

## Additional information

**How to cite this article:** Kjaergaard, M. *et al*. Quantized conductance doubling and hard gap in a two-dimensional semiconductor–superconductor heterostructure. *Nat. Commun.* 7:12841 doi: 10.1038/ncomms12841 (2016).

## Supplementary Material

Supplementary Information Supplementary Figures 1-5, Supplementary Notes 1-2 and Supplementary References
