# Supplementary material for: Quantized conductance doubling and hard gap in a two-dimensional semiconductor–superconductor heterostructure
Source: Nat Commun. 2016 Sep 29;7:12841. doi: 10.1038/ncomms12841 (PMC5056412; doi:10.1038/ncomms12841)
Supplement: Supplementary Information — Supplementary Figures 1-5, Supplementary Notes 1-2 and Supplementary References [file ncomms12841-s1.pdf]

Supplementary Figures

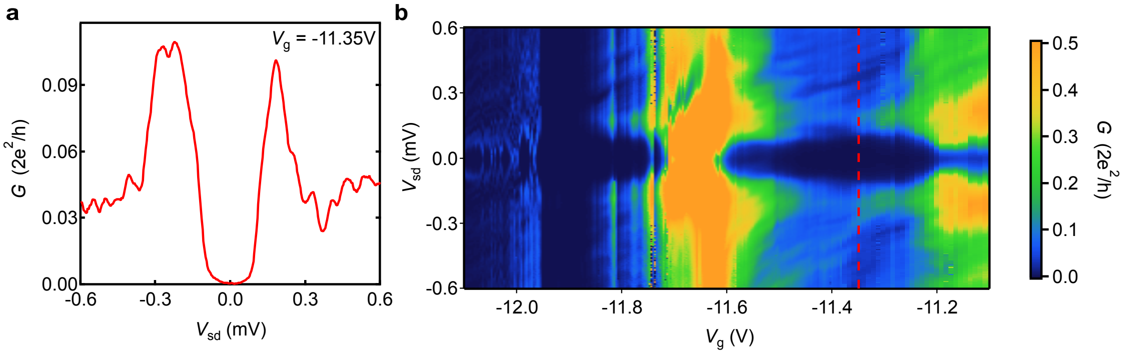

Supplementary Figure 1. **Spectroscopy of the superconducting gap in a wafer with 0 nm InGaAs barrier**. **a**, Differential conductance, G, as a function of source-drain voltage, V_{sd}, in a quantum point contact geometry, with gate voltage $V_{\mathrm{g}} = -11.35$ V. **b**, Differential conductance at finite source-drain voltage, as the split-gate is used to deplete the 2DEG by decreasing V_{g}. Vertical cut in **a** indicated by dashed, red line.

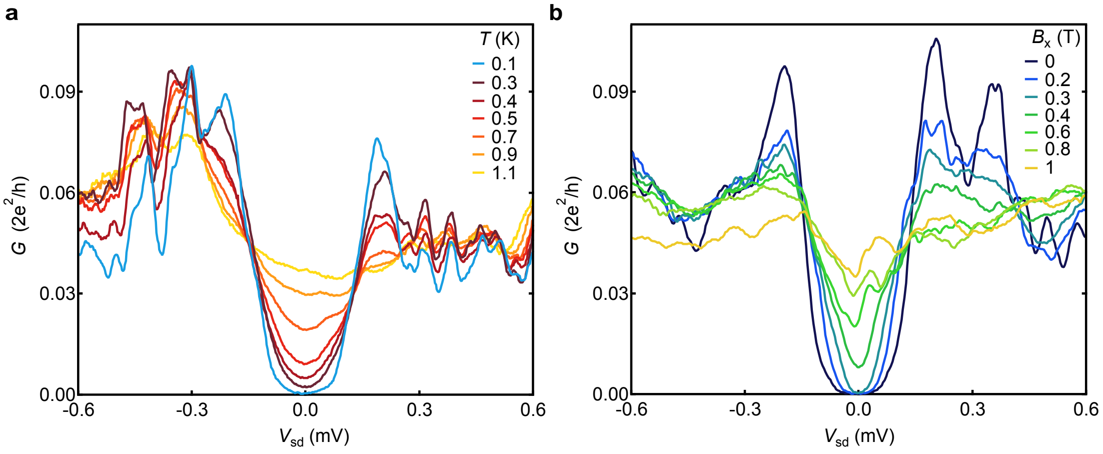

Supplementary Figure 2. **Temperature and magnetic field dependence of the proximity induced superconducting gap**. **a**, Differential conductance as a function of source-drain voltage for several temperatures. **b**, In-plane magnetic field dependence of the superconducting gap (field applied perpendicular to the constriction).

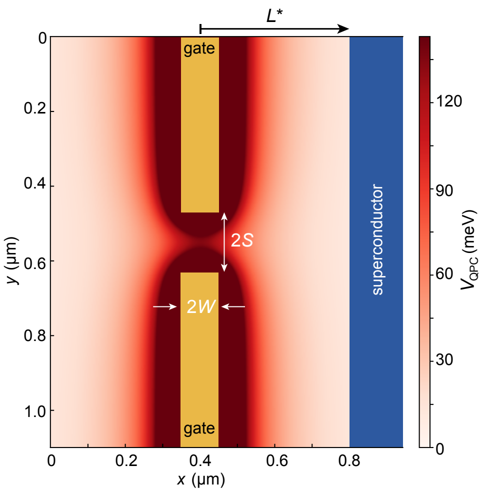

Supplementary Figure 3. **QPC potential layout.** The yellow contours show the geometry of the QPC gates and the red color depicts potential created at the position of 2DEG for $V_\mathrm{g} = -1350$ mV.

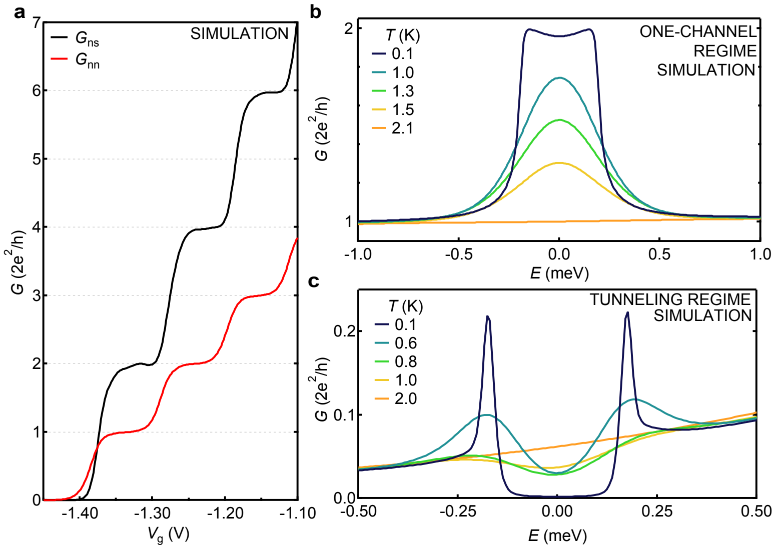

Supplementary Figure 4. **Conductance calculated for a system with $L^* = 230$ nm.** **a** Andreev-enhanced conductance G_{ns} (black curve) and the normal-state conductance G_{nn} (red curve) versus the potential on the QPC gates calculated for $E = 0$. **b** Spectroscopy curves in one-channel regime for $V_\mathrm{g} = -1330$ mV. **c** Tunneling spectroscopy curves for $V_\mathrm{g} = -1408.7$ mV.

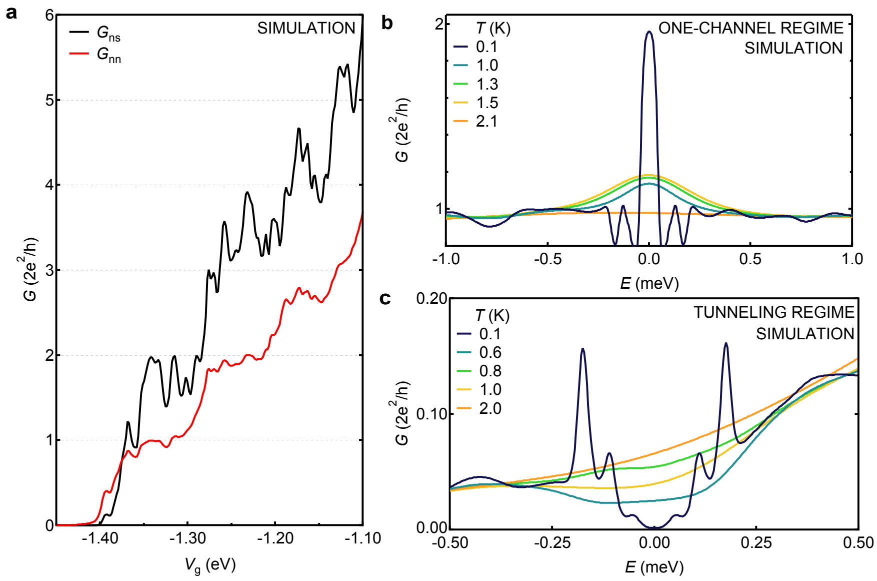

Supplementary Figure 5. **Conductance calculated for a system with $L^* = 800$ nm. a** Andreev-enhanced conductance $G_{\rm ns}$ (black curve) and the normal-state conductance $G_{\rm nn}$ (red curve) versus the potential on the QPC gates calculated for $E = 0$. **b** Spectroscopy curves in the one-channel regime for $V_{\rm g} = -1341$ mV. **c** Tunneling spectroscopy curves for $V_{\rm g} = -1407$ mV.

Supplementary Notes

Supplementary note 1: Measurements on alternate wafer

Under identical growth conditions, a wafer without an InGaAs top barrier (i.e. $b = 0$ nm) between the epitaxial aluminum and the InAs quantum well was produced. The density and mobility, measured using a conventional Hall bar geometry, was $n = 4.5 \cdot 10^{16}$ m^{-2} and $\mu = 4000$ cm^2/Vs, corresponding to a mean free path of $l_e = 150$ nm. In a lithographically similar device to that shown in Fig. 1 of the main text, we observe a hard superconducting gap (Supplementary Figure 1). When the gates are operated in the quantum point contact regime, we did not observe quantized steps in conductance. The non-monotonic decrease in conductance at $V_{\rm sd} = 0$ mV, believed to be due to disorder in the 2DEG, makes the identification of a superconducting gap in this wafer difficult (Supplementary Fig. 1b).

However, by increasing the temperature (Supplementary Fig. 2a) or the magnetic field (Supplementary Fig. 2b) we confirm that the gap in the density of states in Supplementary Fig. 1 is related to the superconducting properties, and not a spurious quantum dot.

Supplementary Note 2: Numerical results

The potential landscape generated by the simulation, used to model the QPC is shown in Supplementary Figure 3. For the simulation we adopt the following parameters: chemical potential $\mu = 143$ meV, mean free path $l_{\rm e} = 230$ nm, effective mass $m^* = 0.05 m_{\rm e}$ (obtained from $k.p$ calculation of the Fermi velocity for a single mode quantum well in the growth direction). We also assume $T_{\rm c} = 1.6$ K and $\Delta^* = 190$ μeV. The QPC geometry is set by the parameters: $W = 50$ nm (width of gates), $S = 75$ nm (separation between gates), L^* (distance from middle of QPC to the superconductor) and $d = 50$ nm (distance from gates down to the 2DEG). We consider a system of the geometry similar to the one presented on Fig. 1b of the main text. Here the superconductor interface is located 230 nm after the QPC.

Supplementary Figure 4a shows the conductance as a function of the gate voltage. The $G_{\rm ns}$ conductance depicted with the black curve is quantized in multiples of $4e^2/h$ as the transport involves transmission of an electron and an Andreev-reflected hole. Supplementary Figures 4b,c show the Andreev-enhanced spectroscopy curves obtained by varying the injection energy E. Supplementary Figures 4b and 4b show, respectively, the calculated finite–bias

properties of the one–channel regime and the tunneling regime, for several values of the temperature. The value of V_g in the simulations are chosen so the conductance at zero bias match the data at $T > T_c$ in Fig. 4 of the main text. The low temperature spectroscopy curves are similar the ones obtained by using the analytic expression of Blonder-Tinkham-Klapwijk (BTK) [1]. However, for energies larger than the gap, the spectroscopy simulations show an increasing trend as a function of E (cf. the orange curves on Supplementary Fig. 4b,c where $T > T_c$), due to an increase of the energy of the injected particle with respect to the QPC potential. This dependence is pronounced in our geometry, because the slopes of the QPC steps are less than 50 meV wide, making the conductance sensitive to changes in E on the scale of single meV.

The low temperature one-channel spectroscopy curve shows maxima at $|E| \simeq \Delta$ (blue curve in Supplementary Fig. 4b) while in the experimental data (cf. Fig. 4a and Fig. 5a of the main text) the curves decrease smoothly as $|V_{sd}|$ is increased. Previous theoretical work [2] showed that the detailed layout of the interface between the normal and superconducting electrodes (at the scale of the coherence length) impacts the subgap conductance due to interference between two electrons tunneling through the interface. Moreover, smearing of the superconducting coherence peak [3] is predicted to be an effect of disorder present in the superconducting film pointing again to the role of normal-superconductor interface.

The experimental structure consist of an extended 2DEG/superconductor interface created by the InGaAs/InAs heterostructure covered by Al. In the present calculations, we are limited to an abrupt semiconductor/superconductor interface. We therefore also consider a case where the distance from the QPC to the interface is increased relative to the lithographic dimensions.

Supplementary Figure 5 shows results obtained for a system with 800 nm distance between the QPC gates and the superconductor interface. In this calculation the scattering region is longer than the mean free path, leading to the peak/dip structures superimposed on the QPC conductance steps. Similar peaks/dips are observed in the experimental data in Figs. 2a,b of the main text. The fluctuations are more pronounced in the superconducting case (G_{ns}) due to the Andreev-enhanced conductance involving traversing the scattering region twice. The resonant features are also visible in the low temperature spectroscopy curves for energies larger the superconducting gap (cf. Supplementary Figs. 5b,c), similar to the experimental curves in Figs. 4 a,c of the main text. Comparable pinch off curves are obtained when the disorder is located before the QPC, if the distance between the QPC and the superconductor are short.

The most notable feature of the system with extended length between the QPC and the superconductor is a significant reduction of the width of the central peak in the one–channel finite–bias simulations (blue curve in Supplementary Fig. 5b). The rapid drop in conductance is a hallmark of an induced gap, for which the chaotic billiard in the region between the QPC gates and superconductor has zero density of states. The energy scale at which conductance drops is denoted E_b, and has the magnitude of Thouless energy [4, 5], and hence it is inversely proportional to the area between the QPC and the interface. For $|E| > E_b$ the billiard has a non-zero discrete spectrum and so for $E_b < |E| < \Delta$ the conductance exhibits oscillations due to transport through resonant states which here are smoothed already for $T = 0.1$ K due to temperature averaging. The smooth resonances are also present in the low-temperature conductance curve in the tunneling regime (Supplementary Fig. 5c).

Supplementary References

[1] Blonder, G. E., Tinkham, M. & Klapwijk, T. M. Transition from metallic to tunneling regimes in superconducting microconstrictions: Excess current, charge imbalance, and supercurrent conversion. *Phys. Rev. B* **25**, 4515–4532 (1982).

[2] Hekking, F. W. J. & Nazarov, Y. V. Subgap conductivity of a superconductor-normal-metal tunnel interface. *Phys. Rev. B* **49**, 6847–6852 (1994).

[3] Feigelman, M. V. & Skvortsov, M. A. Universal Broadening of the Bardeen-Cooper-Schrieffer Coherence Peak of Disordered Superconducting Films. *Phys. Rev. Lett.* **109**, 147002 (2012).

[4] Melsen, J. A., Brouwer, P. W., Frahm, K. M. & Beenakker, C. W. J. Induced superconductivity distinguishes chaotic from integrable billiards. *Europhysics Letters (EPL)* **35**, 7–12 (1996).

[5] Melsen, J. A., Brouwer, P. W., Frahm, K. M. & Beenakker, C. W. J. Superconductor-proximity effect in chaotic and integrable billiards. *Phys. Scr.* **1997**, 223 (1997).
